# Physical activity health literacy in patients with chronic diseases: a concept analysis

**DOI:** 10.3389/fpubh.2025.1673391

**Published:** 2025-10-29

**Authors:** Xiaotian Zhang, Ruixue Bi, Xinyu Wang, Yuxuan Bu, Xiaoping Liu, Yu Liu, Ruihong Cui, Yankang Wang, Hongyan Li

**Affiliations:** ^1^School of Nursing, Jiangxi Medical College, Nanchang University, Nanchang, Jiangxi, China; ^2^Jiangxi Province Key Laboratory of Aging and Disease, Nanchang, Jiangxi, China; ^3^Department of Rehabilitation Medicine, Second Affiliated Hospital of Nanchang University, Nanchang, Jiangxi, China

**Keywords:** health literacy, health behavior, physical activity, concept analysis, chronic disease

## Abstract

**Aims:**

Physical activity health literacy plays a positive role in optimizing individuals' physical activity health behaviors and is one of the key abilities for achieving proactive health management. This study is to clarify the concept of physical activity health literacy in patients with chronic diseases.

**Methods:**

The Rodgers' evolutionary method of concept analysis was used to identify the antecedents, attributes, and consequences of the concept of “physical activity health literacy” in patients with chronic diseases.

**Results:**

Three critical attributes were identified: functional, communicative, and critical physical activity literacy. Antecedents were classified into three categories: disease, cognitive, and social-psychological factors. The consequences include three themes: improving disease symptoms, improving quality of life, and promoting social-psychological health.

**Conclusion:**

The findings of this concept analysis contribute to a deeper understanding and clarification of physical activity health literacy.

## 1 Introduction

Chronic diseases are a leading cause of disease burden (1). Physical inactivity is closely associated with a high incidence of chronic diseases (2). Physical activity effectively prevents chronic non-communicable diseases and alleviates the symptoms of chronic diseases, reduces complications, and improves the quality of life of patients (3). The World Health Organization recommends that patients with chronic diseases should increase their physical activity and alter their sedentary lifestyle to prevent and manage chronic diseases (4).

Good health literacy is essential for maintaining high physical activity levels and establishing healthy exercise habits. Health literacy has become an important indicator of the prevention and control of chronic diseases (5). Low health literacy level in patients with chronic diseases is closely related to poor health behaviors and outcomes (6). Health literacy also affects the development of patients' ability to self-manage their disease (7). Patients with low health literacy typically perform poorly in disease management (8), particularly older adults, for whom low health literacy is highly correlated with the incidence of multiple diseases (9). Higher levels of health literacy are associated with increased participation and persistence rates of physical activity (10).

Presently, many patients with chronic diseases undergoing home rehabilitation have low awareness regarding participation in physical activities; the methods, intensity, volume of physical activity; and risk control of injuries during exercise (11), which affects patients' rational, effective, and continuous participation during physical activity (12). Clinical medical staff have realized the need to improve the level of physical activity awareness among patients with chronic diseases; however, there is still no clear and definite definition of chronic disease physical activity health literacy, which limits the assessment and intervention provided by nurses or other healthcare professionals.

In previous studies, relevant concepts related to physical activity health literacy included “physical activity knowledge,” “exercise knowledge,” and “exercise perceptive.” A majority of the research participants were older adults, adolescents, medical personnel, and fitness coaches. The connotation of the concepts “physical activity knowledge” and “exercise knowledge” emphasizes the depth of understanding related to physical activity or exercises in the context of diseases, and “exercise perceptive” primarily involves patients' awareness of the benefits and barriers of exercise. However, these concepts belong to cognitive terms, they do not involve the application and transformation of physical activity-related knowledge or safe exercise ability, which are essential for patients with chronic diseases.

In some health literacy scales, physical activity-related health literacy is also mentioned. For example, the European Health Literacy Survey Questionnaire (HLS-EU-Q) developed by Sørensen et al. in 2013 (13) includes items related to physical activity, such as “obtaining information on how physical exercise benefits health”; the Student-Athlete Physical Health Literacy Scale developed by Beasley et al. in 2021 (14) further assesses how athletes acquire knowledge about sports nutrition and injury prevention, how they communicate health issues with coaches, and how they critically evaluate training plans and health information.

In 2019, Baek mentioned the concept of “physical activity health literacy”, arguing that Korean children need to develop and improve this type of health literacy, but did not analyze the concept (15). In 2022, Iranian scholars developed the PAHLIO (Physical Activity Health Literacy in Iranian Older Adults) questionnaire, which provides a validated assessment tool for older adults and measures four dimensions: Information Evaluation, Reading Skills, Perception, and Decision-Making (16). Nevertheless, these instruments are not specifically designed for patients with chronic diseases and therefore fail to adequately capture the core dimensions of physical activity health literacy essential for their disease management process. Therefore, clarifying the conceptual connotation of physical activity health literacy for patients with chronic diseases and developing corresponding assessment tools hold significant theoretical and practical significance.

Thus, based on concepts related to health literacy, this study aims to clarify the concept, define attributes, antecedents and consequences related to physical activity health literacy in patients with chronic diseases. Additionally, we aimed to differentiate them from similar concepts, provide a theoretical foundation for the future development of assessment tools, and further enrich the connotations of health literacy. It also provides a basis for formulating physical activity intervention and health education strategies for patients with chronic diseases, so as to promote the improvement of their physical activity health literacy.

## 2 Methods

### 2.1 The process of concept analysis

This study used the Rodgers' evolutionary method of concept analysis (17), which includes six steps: (1) determining the concept to be analyzed, (2) establishing the analysis purpose, (3) defining attributes, (4) determining antecedents and consequences, (5) constructing model cases, and (6) distinguishing related terms.

### 2.2 Data sources

Retrieval was performed using multiple databases, including PubMed, Web of Science, Cochrane, CINAHL, Embase, CNKI (China National Knowledge Infrastructure), and Wanfang. Based on the characteristics of different databases, Boolean logic was applied to search the titles and abstracts of articles, using terms such as “health literacy,” “knowledge,” “physical activity,” and “chronic diseases” for retrieval. The retrieval time range is from the establishment of the database to September 2025. The specific search strategies are detailed in the Supplementary material.

The inclusion criteria were as follows: (1) concepts related to physical activity health literacy, (2) factors related to physical activity health literacy, (3) articles published in Chinese or English. The exclusion criteria were as follows: (1) literatures where the full text was unavailable (e.g., only abstracts provided), (2) literatures irrelevant to the research topic (e.g., those not involving health literacy, physical activity, or chronic disease management), (3) gray literature (e.g., unpublished dissertations, conference abstracts, reports, etc.), (4) studies with duplicate publication or duplicate data.

**Figure 1:**
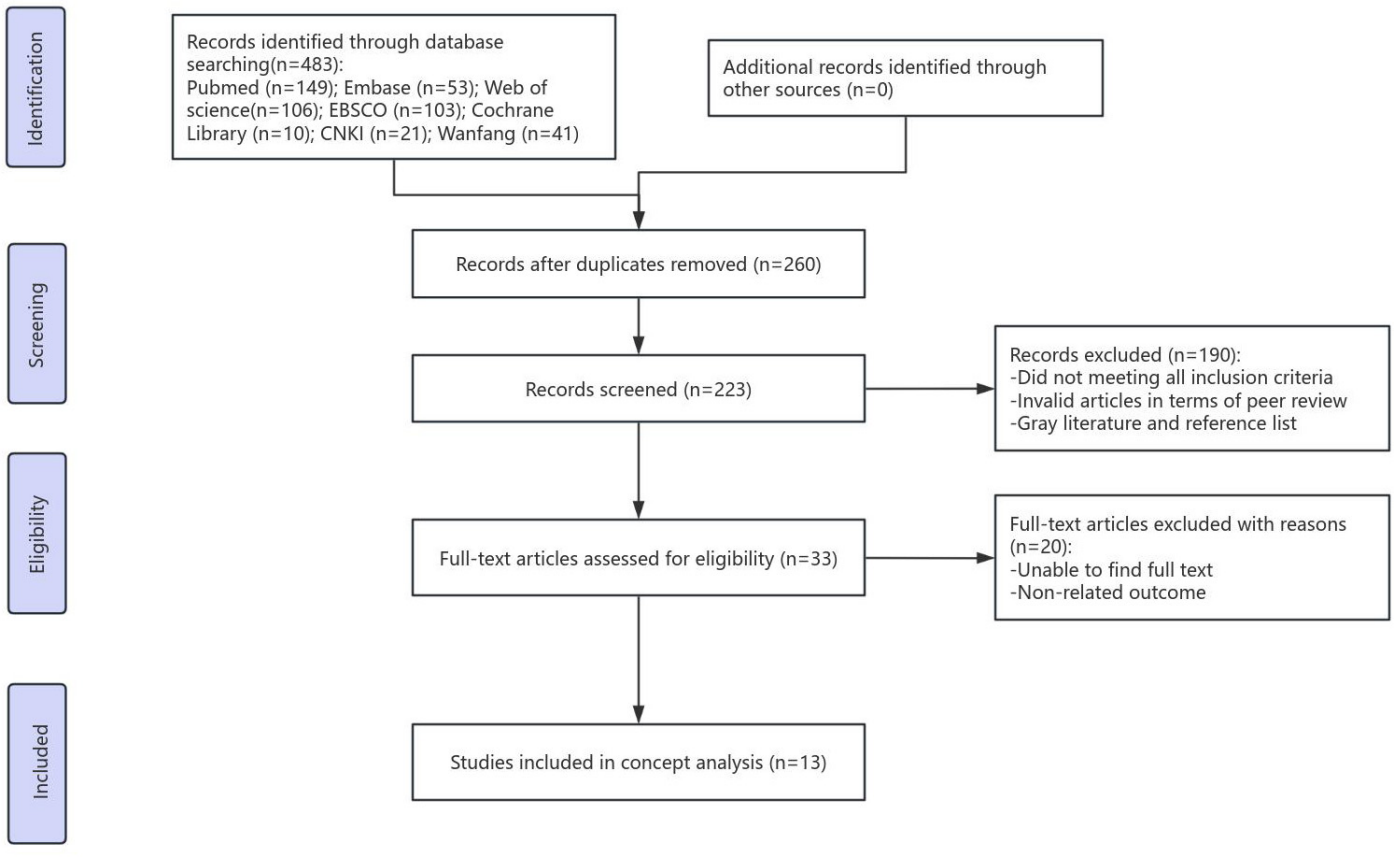
Flow diagram of literature search and selection.

Figure 1 shows the literature retrieval flowchart. The initial database searches yielded 483 records. After removing duplicates, 223 articles remained for screening. The titles and abstracts of these articles were independently screened by two reviewers against the inclusion criteria. Subsequently, the full texts of 33 potentially relevant articles were assessed for eligibility by the same two reviewers. Any disagreements regarding inclusion at each stage were resolved through discussion or, if necessary, by consultation with a third senior reviewer to reach a consensus.

## 3 Results

### 3.1 Evolution of physical activity health literacy

Simonds (18) first proposed the concept of health literacy at the 1974 International Health Education Conference. In 1995, Williams et al. (19) defined health literacy as the ability to complete health-related tasks that require reading and calculation skills. Don (20) suggested that health literacy is a cognitive and social skill that determines individual motivation and the ability to understand and use information to promote and maintain good health. Health literacy is categorized into three dimensions: functional, communicative, and critical health literacy. Functional health literacy means the ability to understand and apply health information to daily life; communicative health literacy refers to the skill of gaining health information from various avenues and applying it to different situations; and critical health literacy refers to the ability to critically analyze health information and use this information to control life events and outcomes. Christina et al. (21) defined health literacy as the range of skills and abilities that individuals use to seek, understand, evaluate, and apply health information and concepts to make informed decisions, reduce health risks, and improve their quality of life.

As the conceptual understanding of health literacy continues to evolve, it has expanded to encompass various branches, such as chronic disease related health literacy, mental health literacy, e-health literacy, nutrition health literacy, medication health literacy and so on. In 2017, the International Physical Literacy Association described physical literacy as “the motivation, confidence, physical ability, knowledge, and understanding of individuals to participate in physical activities” and engage in sports activities as a lifelong habit (22). In 2019, Baek and Lee (15) first introduced the term “physical activity health literacy” and outlined it as “the overall ability to promote and maintain a healthy life in terms of function, cognition, and emotion, and to continuously participate in physical activities”. However, it does not take into account the patients with chronic diseases.

### 3.2 Differentiating related terms

#### 3.2.1 Physical literacy

As articulated by Whitehead (23), physical literacy is not merely “physical ability” or “motor skill,” but rather a holistic human capacity integrating physical, cognitive, and emotional dimensions, rooted in existential and phenomenological perspectives that emphasize the embodied nature of human existence. It represents the “root” of an individual's lifelong engagement with the world through movement, encompassing motivation, confidence, physical competence, knowledge, and understanding of physical activity. Caldwell et al. further elaborate that physical literacy includes the emotional, cognitive, behavioral, and physical elements of an individual's participation in physical activities, reflecting a comprehensive and foundational capability to interact with diverse environments (24).

#### 3.2.2 Physical education core literacy and sport literacy

“Physical education core literacy” refers to a set of key abilities and qualities that individuals possess in physical activities, including athletic ability, health behaviors, and sports morality (25). It serves as the “cultivation blueprint,” forming the educational framework and goals for schools to systematically develop students' physical and related competencies.

Pill reflected on traditional teaching methods and proposed sports literacy from a multi-literacy perspective, aiming to make it the goal of sports education (26). Yong-Hui et al. noted that sports literacy is a type of sports quality developed through the combination of innate natural factors and acquired social influences, encompassing theoretical, appreciative, methodological, and coaching knowledge related to sports (27). Sport literacy can thus be viewed as a “branch,” highlighting professional skills and cultural understanding within specific sports.

The differences between these concepts—physical activity health literacy, physical literacy, physical education core literacy, and sport literacy—are presented in Table 1.

**Table 1:** The differences among the concepts of physical activity related health literacy.

**Literacy type**	**Core focus**	**Goal orientation**	**Target audience**
Physical activity health literacy	Health promotion and disease prevention	Personal and public health: accessing, understanding, and applying knowledge about physical activity to make health-beneficial choices and changes	The general public, especially people with chronic diseases and those at risk of health issues
Physical literacy	Comprehensive personal development and lifelong engagement	Individual agency: developing motor competence, confidence, motivation, and knowledge to enjoy physical activity lifelong in diverse environments	Children and adolescents
Physical education core literacy	Goals and outcomes of school education	Educational objectives: the comprehensive manifestation of students' key competencies, essential character, and values that should be achieved through physical education courses	Students in school
Sport literacy	Competence in specific sports	Sports performance and cultural understanding: the ability to effectively participate in specific sports, understand their rules, strategies, and culture	Athletes, coaches, and sports enthusiasts

### 3.3 Defining attributes of physical activity health literacy

Table 2 presents the attributes, antecedents, and consequences of the included studies. Table 3 presents the content descriptions of each attribute in the included studies. Through a literature analysis, the defining attributes of physical activity health literacy were extracted, including three core attributes: functional, communicative, and critical physical activity health literacy.

**Table 2:** Characteristics of the studies included.

**Article**	**Study country**	**Design**	**Population**	**Attributes**	**Antecedents**	**Consequences**
				•**Functional (F)** •**Communicative (C)** •**Critical (CR)**	•**Disease (D)** •**Cognitive (C)** •**Social-psychological (S)**	•**Disease symptoms (DS)** •**Life quality (LQ)** •**Social-psychological health (SH)**
Buja et al. (59)	Italy	Review	-	F; C; CR	C	DS
Babak et al. (16)	Iran	Scale validation study	Older adults	F; C	C; S	DS
Beasley et al. (14)	USA	Empirical research	Students	F; C; CR	C; S	SH
Zhang and Li (37)	China	Scale validation study	Adults	F; C; CR	C	LQ
de Boer et al. (60)	The Netherlands	A qualitative study	Patients with a chronic disease	F; C; CR	D; C; S	DS
Sheshadri et al. (30)	USA	A qualitative study	Patients and their care partners	F; C; CR	D; C	DS; LQ
Sørensen et al. (13)	The Netherlands	Scale validation study	-	F; C; CR	C; S	LQ
Gunnell et al. (61)	Canada	Scale validation study	Children	F	C	SH
Liu et al. (5)	China	Empirical research	Adults	F; C; CR	C	DS
Berkman et al. (6)	UK	Review	-	F	C	DS
McCormack et al. (62)	Ireland	A qualitative study	Pulmonary hypertension patients	F; C; CR	D	DS; LQ
Wang et al. (63)	China	Cross-sectional survey	Older adults	F; C; CR	C	DS
Whitehead (23)	UK	Review	-	F; C; CR	C	SH

**Table 3:** Description of the attributes of the included studies.

**Categories**	**Description**
Functional	Mastering and understanding health knowledge related to physical activity [11 articles: (5, 6, 13, 14, 23, 30, 37, 59, 61–63)] Interest and motivation [3 articles: (30, 61, 62)] Exercise confidence [3 articles: (23, 30, 61)] Awareness of the importance of health behaviors [4 articles: (5, 16, 37, 60)]
Communicative	Economic support [3 articles: (5, 13, 60)] Family and peer support [2 articles: (30, 61)] Successful experiences [2 articles: (23, 60)] Individualized exercise programs [1 article: (60)] External supervision and feedback [4 articles: (14, 30, 59, 62)] Good compliance [4 articles: (13, 16, 62, 63)]
Critical	Self-monitoring and risk prevention [5 articles: (5, 13, 37, 59, 62)] Reflecting and questioning health issues [2 articles: (23, 30)] Self-management: Dealing with temporary interruptions due to physical discomfort or seasonal factors [3 articles: (14, 60, 63)]

#### 3.3.1 Functional physical activity health literacy

Functional physical activity health literacy refers to an individual's ability to read and understand physical activity-related knowledge and attitudes. This attribute is grounded in the foundational dimensions of Physical Literacy as described by Whitehead, which include the knowledge, understanding, motivation, and confidence necessary for physical engagement (23). It is manifested as patients being able to actively read physical activity-related knowledge, being aware of the importance of physical activity in promoting health and disease recovery, understanding the exercise requirements, having an interest in participating in physical activity, and intending to develop regular physical activity habits. Zhang et al. (28) noted that physical activity cognition includes three core categories: physical activity knowledge (basic knowledge and the value of participating in physical activity), attitude, and intention. Therefore, functional physical activity health literacy operationalizes key components of Physical Literacy by focusing specifically on health-related knowledge acquisition, motivation for engagement, and the confidence to maintain activity, all of which are essential for initiating and sustaining health-promoting behaviors among patients with chronic diseases.

#### 3.3.2 Communicative physical activity health literacy

Communicative physical activity health literacy is an individual's ability to actively acquire, communicate, and exchange health information related to physical activity with healthcare professionals or others, as well as the ability to use this information to participate in clinical decision-making. This attribute reflects the interactive and relational dimension of Physical Literacy, which emphasizes the role of the embodied self in communicating and connecting with others and the environment (23). Individuals can actively acquire, learn, and communicate health information related to physical activity from medical personnel, family members, fellow patients, and other channels through written, oral, or audiovisual materials, and can actively communicate their experiences of physical activity, obtain beneficial health information, and participate in clinical decision-making. Laird et al. (29) noted out that social support has a positive effect on exercise behavior, and receiving high levels of social support and encouragement has a significant motivating effect on the exercise levels of individuals. Sheshadri et al. (30) conducted a family-based activity plan focusing on health education related to exercise for patients with chronic kidney disease and their families, emphasizing the integration of physical activity into the daily lives of patients. The results revealed a significant increase in patient persistence and participation. When patients observe a successful experience among fellow patients, they are more probable to actively engage in physical activity (31). Thus, communicative physical activity health literacy extends the concept of Physical Literacy into the social and collaborative context of health management, highlighting the importance of shared knowledge and supportive interactions.

#### 3.3.3 Critical physical activity health literacy

Critical physical activity health literacy is the ability of an individual to analyze, evaluate, and apply physical activity-related information. It typically manifests as patients' ability to rationally analyze and correctly evaluate the reliability and scientific validity of physical activity-related knowledge; being able to choose appropriate types, intensities, and amounts of individual physical activity based on their conditions and personal tolerance; understanding contraindications; and having the ability to deal with adverse events related to physical activity or prevent risks and manage physical activity autonomously. This attribute aligns with Whitehead's emphasis on the intelligent and adaptive application of physical capacities, where individuals not only respond to environments but also critically assess and innovate in their movement responses (23). For example, patients with coronary heart disease may find that high-intensity exercise can improve heart function after reading exercise guidelines. However, patients should be aware that high-intensity exercise may also increase the risk of malignant cardiovascular events, such as sudden cardiac death and myocardial infarction (32); thus, they should adjust their exercise plan according to their disease condition to ensure safe exercise. Critical physical activity health literacy thus represents the highest order of physical engagement, integrating judgment, adaptation, and self-regulation—core elements of Physical Literacy—into the context of health-specific decision-making and behavior.

### 3.4 Antecedents of physical activity health literacy

**Figure 2:**
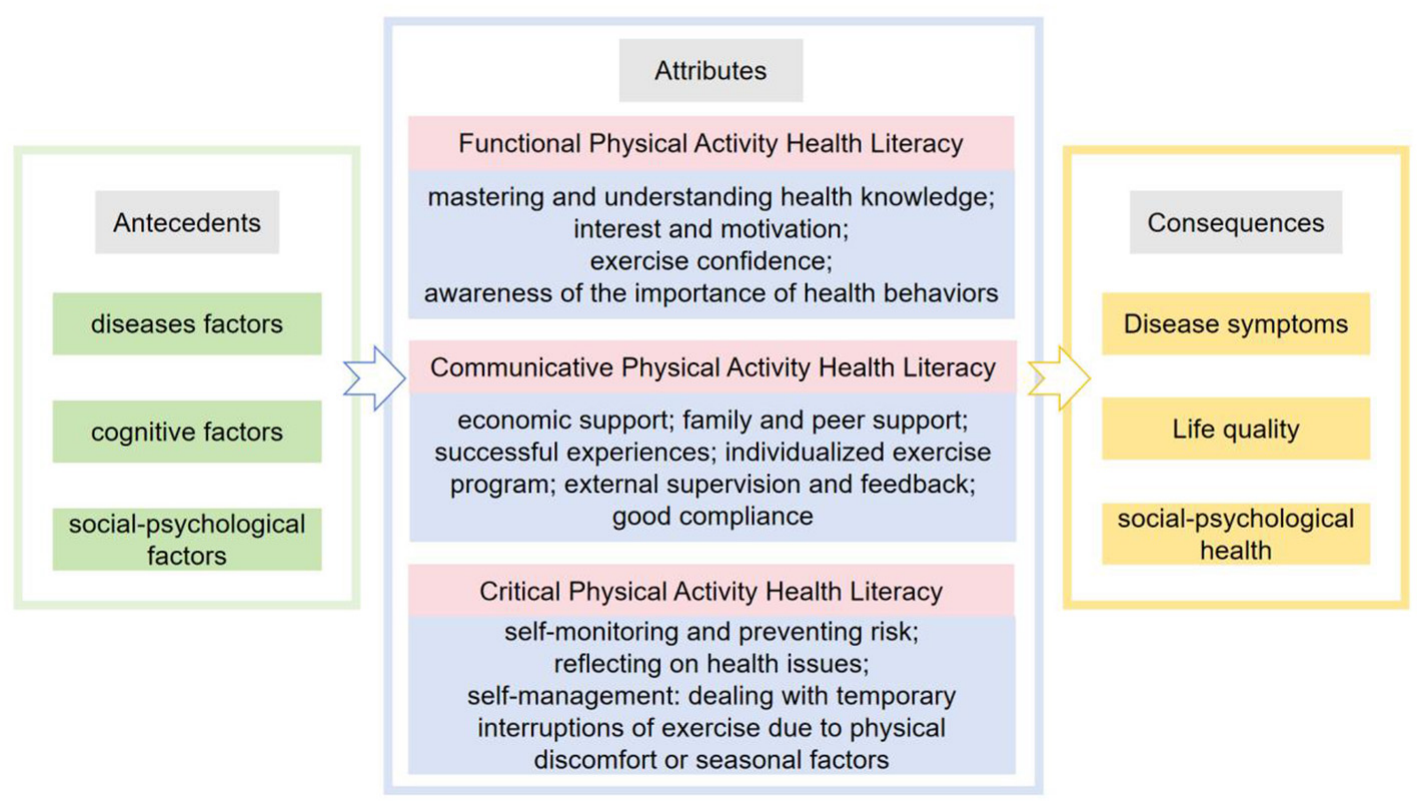
The antecedents, attributes, and consequences of physical activity health literacy.

The antecedents of physical activity health literacy include disease, cognitive, and social-psychological factors (Figure 2).

#### 3.4.1 Disease factors

Physical activity health literacy is affected by disease factors such as restricted physical function, complications, pain, fatigue, and frailty. Patients with chronic diseases often experience varying degrees of complications that limit their physical function, leading to a loss of the willingness to engage in physical activity. For example, patients with chronic low back pain are limited in physical activity owing to musculoskeletal pain (33), and patients with rheumatoid arthritis are reluctant to engage in physical activity owing to fatigue during exercise (34). Depression (35) and stroke can cause decreased cognitive function and memory (36), thereby affecting the ability to engage in physical activity.

#### 3.4.2 Cognitive factors

Physical activity cognition is the basis and prerequisite for physical activity-related behaviors. A lack of exercise information is a major barrier to patient participation in physical activities (30). Without knowledge of physical activities, behavior becomes a mechanical repetition of actions, hindering the development of a positive attitude and intention toward physical activities and leading to a greater possibility of losing interest (37). Understanding physical activity-related knowledge helps patients recognize the value and benefits of physical activity in disease recovery (30). Moreover, patients with higher education levels are more likely to actively seek social support resources that are beneficial for disease recovery and are more receptive to health-related knowledge, thereby establishing health beliefs and behaviors (38).

#### 3.4.3. Social-psychological factors

Psychological stress, negative emotions, insufficient social support, and poor economic conditions hinder physical activity health literacy in patients. Physical activity health literacy is positively associated with positive emotions and negatively associated with negative emotions (39). Psychological stress leads to reduced physical activity and increased sedentary behavior (40). Engaging in appropriate and challenging exercises can enhance confidence and increase motivation to engage in physical activity. Sheshadri et al. (30) noted that many patients with chronic kidney disease require a certain level of support from their peers to perform exercises. Insufficient social support causes negative emotions and low levels of physical activity (28). Additionally, economic status is positively correlated with health literacy (41). People living in less-developed areas have lower rates of physical activity than those living in more-developed areas (42).

### 3.5 Consequences of physical activity health literacy

The consequences of physical activity-related health literacy include improved disease symptoms, enhanced quality of life, and promoted social-psychological health (Figure 2).

#### 3.5.1 Disease symptom improvement

Enhanced physical activity health literacy is reflected in high exercise participation rates and good compliance (10). Improved participation and compliance with physical activities contribute to improvement in patients' disease symptoms. Studies have shown that maintaining moderate or vigorous exercise for up to 10 years reduces the incidence of diabetes by 49% (43). In a randomized controlled trial of patients with heart failure (44), 72.7% of the patients considered that exercise could improve their physical health (endurance, activity, and energy); however, these effects depended on the patient's exercise compliance. A comprehensive exercise plan cannot demonstrate its value without adherence.

#### 3.5.2 Enhancing the quality of life of patients

Increased frequency of physical activity or decreased sedentary time resulted in better health-related quality of life of patients (45). Improved fatigue and sleep, reduced readmission rates, alleviation of disease progression, promotion of recovery, and reduction in complications were observed in the patients. Effective interventions for physical activity can improve cancer-related fatigue in patients (46). Physical activity can effectively improve the sleep quality of older adults with chronic diseases (47).

#### 3.5.3 Promoting social-psychological health

Improvement in physical activity health literacy promotes participation in physical activities, thereby bringing about positive physiological and social-psychological adaptations and improving physical, psychological, and social health (24). Denche-Zamorano et al. (48) noted that exercise could increase the secretion of endorphins, thereby reducing depression and anxiety, improving self-esteem, and alleviating mental suffering. A systematic review (49) showed that physical training could significantly improve the depression status of patients undergoing dialysis.

### 3.6. Model cases of physical activity health literacy

#### 3.6.1 Negative physical activity health literacy

Mr. Wang, a 52-year-old man with a junior high school education, was a patient with coronary heart disease who underwent percutaneous coronary intervention. He typically did not actively read exercise-related information for coronary heart disease. During the exercise, he felt chest tightness but did not communicate with the doctor on time. The pain was relieved after rest; however, he later stopped exercising because of fear of recurrence. During follow-up, medical personnel observed that his blood pressure was unstable, and his weight was too high, which increased the risk of cardiovascular disease. He was advised to develop good physical activity habits, mainly including aerobic exercise, 5 days a week, lasting more than 30 min each time. Thereafter, Mr. Wang often visited the gymnasium for activities and participated in resistance training. During one exercise, he experienced intensification of chest tightness, difficulty in breathing, palpitations, and angina pectoris and was hospitalized for treatment. The physician's diagnosis revealed that the patient's myocardial oxygen consumption and blood supply were insufficient owing to excessive exercise, which increased the burden on the heart and exacerbated the condition.

Mr. Wang's case shows that his low educational level led to low cognitive function and that he did not actively acquire exercise knowledge, indicating low functional physical activity health literacy. When encountering problems with exercise, he did not communicate with his doctor on time, mistakenly believing that he was not suitable for physical activity. Therefore, he failed to form a good exercise habit, indicating low communicative physical activity health literacy. Mr. Wang later participated in the exercise; however, he exercised blindly and adopted the wrong exercise method. Excessive exercise led to insufficient myocardial oxygen consumption and blood supply, increasing the burden on the heart and exacerbating the condition, indicating that Mr. Wang's critical physical activity health literacy was low. Therefore, Mr. Wang is at risk of worsening his disease condition and having a declining quality of life and poor mental state.

#### 3.6.2 Positive physical activity health literacy

Mr. Li, a 46-year-old man with a bachelor's degree, is an office worker who sits for long periods, has type 2 diabetes, is currently overweight, and is undergoing medical treatment. Mr. Li is enthusiastic about reading health-related books. After the illness, he actively adhered with the treatment plan and communicated with medical personnel about his condition. With the help of medical personnel, he developed a personalized exercise plan that combined aerobic exercises (brisk walking, jogging, and swimming) and strength training. He engaged in at least 150 min of moderate-intensity aerobic exercise per week and performed two strength training sessions. Owing to the risk of hypoglycemia during exercise in patients with diabetes, his friends advised him to stop training daily and rest. However, after reading books, Mr. Li understood the importance of exercise and continued to adhere to regular exercise. He regularly visited the hospital for follow-up, and medical personnel advised him to consume medication on time and regularly monitor his blood glucose levels. After consistently engaging in exercise, Mr. Li's blood glucose levels remained normal, and his weight decreased without any complications or adverse outcomes.

Mr. Li had higher education levels, indicating better cognitive function. He continues to actively read health-related books and demonstrates good functional physical activity health literacy. He communicated actively with medical personnel, developed personalized exercise plans, actively participated in physical activities, and formed good exercise habits, indicating good communicative physical activity health literacy. His friends advised him to stop exercising because of the risk of hypoglycemia in diabetes; however, he obtained correct health knowledge through books and adopted appropriate exercise methods, eventually managing his blood glucose level within a stable range without complications or adverse outcomes, indicating good critical physical activity health literacy. Thus, with good physical activity health literacy, Mr. Li's overall health has improved, and he can prevent potential health risks.

## 4 Discussion

### 4.1 Conceptual definition and summary of attributes

This study employs Rodgers' Evolutionary Concept Analysis to clarify the concept of Physical Activity Health Literacy in patients with chronic diseases (17, 50). Based on the concept analysis, physical activity health literacy comprises three defining attributes: functional, communicative, and critical. It is defined as the ability of patients to acquire, comprehend, interpret, and apply physical activity-related information, coupled with the capacity to actively engage in clinical decision-making to enhance health outcomes. The antecedents of physical activity health literacy encompass disease-related, cognitive, and social-psychological factors. Its primary consequences include improvement in disease status, enhancement of quality of life, and promotion of social-psychological health.

### 4.2 Differentiation from related concepts

Both physical education core literacy and sport literacy are primarily applied in educational and specific sports contexts, emphasizing athletic skills, knowledge, adaptability, and ethical behavior. Physical activity health literacy, however, transcends these boundaries. It focuses on how individuals engage in physical activity, overcome challenges, and emphasizes scientific and health-related aspects. Its core aim is to enhance physical fitness, prevent disease, and improve overall health through appropriate activity. For patients with chronic diseases, it entails understanding physical activity concepts, interacting with healthcare providers, making informed decisions, and developing essential self-management skills.

Physical literacy provides the foundational framework for all three attributes of physical activity health literacy. Physical activity health literacy is conceptualized as the “fruit”—a competency explicitly oriented toward health outcomes. It represents the deepening and application of physical literacy within the realm of health management, particularly for chronic diseases. This framework integrates the functional, communicative, and critical dimensions from health literacy theory with the holistic, lifelong participation in physical activity advocated by physical literacy. While physical literacy provides the foundational motivation, confidence, and physical competence, it falls short of addressing the complex health-specific demands faced by individuals with chronic conditions. Physical activity health literacy specifically channels these foundational capacities to meet these needs: the communicative attribute enables patients to interact effectively with healthcare providers to tailor exercise plans, while the critical dimension highlights the paramount importance of safety and self-regulation, requiring patients to continuously evaluate activities based on their disease status. Together, these elements form a comprehensive framework that promotes health and effectively manages risk.

### 4.3 In-depth discussion of attribute dimensions

Don suggested that functional health literacy is the ability to understand health information and the willingness to use this information to enhance health behaviors (20). Davis et al. (51) asserted that functional health literacy is the ability to understand health terminology, while Lee et al. (52) indicated that it refers to the ability of individuals to read and assess health information. Reading and understanding health information related to physical activity are prerequisites for participation in physical activity. Patients with cognitive impairment or low literacy may not understand the benefits of physical activity, affecting their motivation to engage in it (37). Therefore, functional physical activity health literacy in patients with chronic diseases should include physical activity awareness, interest, and motivation. Functional physical activity health literacy effectively channels the inherent human capacities for movement and understanding, as outlined in Physical Literacy, into the health context, emphasizing the knowledge and motivation required to initiate health-enhancing physical activities.

Don noted that communicative health literacy refers to the ability to obtain and apply health information using various methods and changing contexts (20). Wallace et al. (53) asserted that communicative health literacy is the patient's ability to use medical services. Massey et al. (54) suggested that communicative health literacy should include doctor-patient communication, interpersonal interaction, and application of health information. Active participation in doctor-patient interactions helps patients to completely understand their conditions and treatment plans. Patients and medical professionals collaborate to develop personalized physical activity plans and increase participation in physical activity. Timely feedback on the effects and adverse reactions of exercise facilitates the appropriate alteration of physical activity plans (55).

In addition to face-to-face interaction, information exchange is crucial for doctor-patient interaction. Currently, the application of current technology, including Internet health management platforms, telemedicine, and social media, such as WeChat, has effectively promoted doctor-patient communication and helped improve the treatment outcomes of chronic diseases (56). Additionally, the support of family and peers (30) and the successful experiences of fellow patients (37) can also motivate patients to exercise. Our findings on communicative physical activity health literacy extend Whitehead's emphasis on the embodied self as a medium for interaction. Therefore, communicative physical activity health literacy in patients with chronic disease should include the ability of patients to communicate and apply exercise-related information with physicians, family members, and fellow patients, and to receive appropriate support and feedback.

Don noted that critical health literacy is the ability to critically analyze health information and use it to control life events and emergencies (20). Sykes et al. (57) have reported that the potential benefit of critical health literacy was the improvement of health outcomes. Studies have confirmed (41) that critical health literacy is more important than functional health literacy in the management of chronic diseases. Chronic diseases typically cause weakness and disabilities. When participating in physical activity, patients should have the ability to prevent adverse outcomes, injury, exacerbation of disease due to injury, and cessation of activity, and to ensure safety and sustainability of physical activity. For example, patients with stroke should avoid causing misuse syndrome due to improper activities. Patients with chronic disease should learn to monitor key health indicators, such as heart rate and blood pressure, before and after exercise. They should accurately record and analyze these results for effective communication and discussions with their physicians. Providing self-management skills to patients is more efficient in improving clinical outcomes than only offering the information (58). A self-management education plan for patients with chronic diseases can improve treatment outcomes and reduce treatment costs. Therefore, the ability of patients to identify and use physical activity health information for preventing adverse outcomes and self-management should be part of the connotations of critical physical activity health literacy. The critical attribute of physical activity health literacy strongly aligns with Whitehead's depiction of the physically literate individual who “reads” the environment and responds intelligently. For patients, this “environment” includes their own body and disease status; critical physical activity health literacy is the ability to analyze information critically and adapt exercise plans accordingly, embodying the highest form of self-regulation and applied intelligence in health management.

### 4.4 Practical implications and clinical application

Healthcare professionals can incorporate the physical activity health literacy assessment into routine evaluations. By understanding patients' specific proficiency levels across the three dimensions—functional, communicative, and critical—they can develop personalized health education and behavioral intervention plans. For example, for patients with insufficient functional physical activity health literacy, the focus should be on providing basic knowledge and motivating them; for those with inadequate communicative physical activity health literacy, efforts should be made to enhance doctor-patient communication and build social support networks; and for patients lacking critical physical activity health literacy, emphasis should be placed on training in risk identification and self-management skills.

## 5 Limitations

Currently, research on physical activity health literacy is in its infancy, with relatively few studies, and the definition of this concept needs to be gradually improved. Additionally, existing research mainly focuses on students and older adults, and lacks targeted research on different populations with chronic diseases. Future research should expand to different chronic disease populations to understand the physical activity health literacy levels and influencing factors in these populations.

## 6 Conclusion

Conceptually grounded in the dimensions of health literacy, this study establishes a conceptual framework for Physical Activity Health Literacy by defining its core attributes, antecedents, and consequences through Rodgers' evolutionary concept analysis. This concept analysis formulates a quantifiable framework for clinical practice by integrating physical activity with the practical requirements of chronic disease management. The framework thereby supports healthcare professionals in the development of physical activity health literacy scales and the design of tailored interventions and educational programs to promote physical activity participation among chronic disease patients. It is not only of great significance for the management of chronic diseases at the individual level but also demonstrates broad application prospects in the context of global public health.

## Data Availability

The original contributions presented in the study are included in the article/Supplementary material, further inquiries can be directed to the corresponding author.
