# Supplementary material for: Physical activity health literacy in patients with chronic diseases: a concept analysis
Source: Front Public Health. 2025 Oct 29;13:1673391. doi: 10.3389/fpubh.2025.1673391 (PMC12605064; doi:10.3389/fpubh.2025.1673391)
Supplement: Supplementary file 1 [file Data_Sheet_1.PDF]

Supplementary Material

Databases	Details
Cochrane (10)	("health literacy" OR "health knowledge" OR "health attitude" OR "health behavior" OR "health belief" OR "health perception" OR "health awareness")<br>AND ("physical activity" OR exercise OR "physical exercise")<br>AND ("chronic disease" OR "chronic diseases" OR "chronic illness" OR "long term conditions" OR "noncommunicable diseases")<br>AND ("systematic review" OR "literature review" OR "narrative review" OR "concept analysis" OR "scoping review" OR "scale" OR "qualitative study")<br>in Title Abstract Keyword
PubMed (149)	("health literacy"[MeSH Terms] OR "health knowledge"[MeSH Terms] OR "health attitude"[MeSH Terms] OR "health behavior"[MeSH Terms] OR "health belief"[Title/Abstract] OR "health perception"[Title/Abstract] OR "health awareness"[Title/Abstract]) AND ("physical activity"[MeSH Terms] OR "exercise"[MeSH Terms]) AND ("chronic disease"[MeSH Terms] OR "chronic illness"[Title/Abstract] OR "long term conditions"[Title/Abstract] OR "noncommunicable diseases"[MeSH Terms]) AND (review[Publication Type] OR "systematic review"[Publication Type] OR "literature review"[Title/Abstract] OR "narrative review"[Title/Abstract] OR "concept analysis"[Title/Abstract] OR "scoping review"[Title/Abstract]) OR "scale"[Title/Abstract]) OR "qualitative study"[Title/Abstract])
Embase (53)	('health literacy'.ti,ab,kw OR 'health knowledge'.ti,ab,kw OR 'health attitude'.ti,ab,kw OR 'health behavior'.ti,ab,kw OR 'health belief'.ti,ab,kw OR 'health perception'.ti,ab,kw OR 'health awareness'.ti,ab,kw)<br>AND ('physical activity'.ti,ab,kw OR exercise.ti,ab,kw OR 'physical exercise'.ti,ab,kw)

	AND ('chronic disease'.ti,ab,kw OR 'chronic diseases'.ti,ab,kw OR 'chronic illness'.ti,ab,kw OR 'long term conditions'.ti,ab,kw OR 'noncommunicable disease'.ti,ab,kw)<br>AND ('systematic review'.ti,ab. OR 'literature review'.ti,ab. OR 'narrative review'.ti,ab. OR 'concept analysis'.ti,ab. OR 'scoping review'.ti,ab. OR "scale".ti,ab. OR"qualitative study".ti,ab.)
Web of Science (106)	(TS=("health literacy" OR "health knowledge" OR "health attitude" OR "health behavior"<br>OR "health belief" OR "health perception" OR "health awareness")<br>AND TS=("physical activity" OR exercise OR "physical exercise")<br>AND TS=("chronic disease*" OR "chronic illness*" OR "long term condition*" OR "noncommunicable disease*"))<br>AND (TS=("literature review" OR "systematic review" OR "narrative review" OR "concept analysis" OR "scoping review" OR "integrative review" OR "scale" OR "qualitative study"))
CINAHL (103)	((MH "Health Literacy") OR (MH "Health Knowledge") OR (MH "Health Attitudes") OR (MH "Health Behavior")<br>OR TI "health literacy" OR TI "health knowledge" OR TI "health attitude" OR TI "health behavior" OR TI "health belief" OR TI "health perception" OR TI "health awareness")<br>AND ((MH "Exercise") OR (MH "Physical Activity") OR TI "physical activity" OR TI exercise OR TI "physical exercise")<br>AND ((MH "Chronic Disease") OR (MH "Noncommunicable Diseases") OR TI "chronic disease" OR TI "chronic diseases"<br>OR TI "chronic illness" OR TI "long term conditions" OR TI "noncommunicable disease")<br>AND (PT review OR PT systematic review OR TI "literature review" OR TI "systematic review" OR TI "narrative review" OR TI "concept

	analysis" OR TI "scoping review" OR TI "scale" OR TI "qualitative study")
CNKI(21)	(主题=健康素养 + 健康知识 + 健康态度 + 健康行为 + 健康信念 + 健康认知 + 健康意识)<br>AND (主题=体力活动 + 运动 + 体育锻炼 + 身体活动 + 体育活动)<br>AND (主题=慢性病 + 慢性疾病 + 长期病症 + 非传染性疾病)<br>AND (摘要=综述 + 文献综述 + 系统评价 + 概念分析 + 量表 + 质性研究)
Wanfang(41)	(主题:("健康素养" OR "健康知识" OR "健康态度" OR "健康行为" OR "健康信念" OR "健康认知" OR "健康意识")<br>AND 主题:("体力活动" OR "运动" OR "体育锻炼" OR "身体活动" OR "体育活动")<br>AND 主题:("慢性病" OR "慢性疾病" OR "长期病症" OR "非传染性疾病")<br>AND 摘要:("综述" OR "文献综述" OR "系统评价" OR "概念分析" OR "量表" OR "质性研究")
